# Health and environmental co-benefits of reducing ultra-processed food consumption in Argentina: a modelling study

**DOI:** 10.1088/1748-9326/aea587

**Published:** 2026-09-17

**Authors:** Christian García-Witulski, Mariano Rabassa, Oscar Melo, Juliana Helo Sarmiento, Valentina Strappa

**Affiliations:** 1 Centro de Desarrollo Humano Sostenible, Pontificia Universidad Católica Argentina, Buenos Aires, Argentina; 2 Centro Latinoamericano de Excelencia en Cambio Climático y Salud, Universidad Peruana Cayetano Heredia, Lima, Perú; 3 Facultad de Emprendimiento, Negocios y Economía, Universidad Espíritu Santo, Samborombón, Ecuador; 4 Centro Interdisciplinario de Cambio Global, Pontificia Universidad Católica de Chile, Santiago, Chile; 5 Departamento de Economía Agraria, Facultad de Agronomía y Sistemas Naturales, Pontificia Universidad Católica de Chile, Santiago, Chile; 6 Facultad de Economía, Universidad de los Andes, Bogotá, Colombia

**Keywords:** ultra-processed foods, mortality, comparative risk assessment, economic costs, environmental impacts, Argentina

## Abstract

Reducing ultra-processed food (UPF) consumption is widely proposed to improve population health, but whether it also generates environmental co-benefits depends on the foods that replace UPFs in the diet. We developed a comparative risk assessment macrosimulation using nationally representative dietary data from Argentina to estimate the mortality and economic burden attributable to UPF consumption and to evaluate policy-relevant reduction scenarios. The scenarios reduced UPF exposure without replacement or replaced the displaced dietary energy or food mass using alternative baskets, including one aligned with the National Dietary Guidelines. We also decomposed the environmental effects of the guideline-concordant basket by food group. In 2019, UPF consumption was associated with 9823 premature deaths, 349 686 years of life lost, and 0.84 billion international dollars in indirect costs. Under the most ambitious reduction target, alternative replacement baskets averted the same 8747 deaths but produced markedly different environmental outcomes, ranging from improvements in all four environmental indicators to increases in all four. The guideline-concordant basket increased water use and eutrophication while producing little change in greenhouse gas emissions and land use. The food-group decomposition showed that these aggregate outcomes reflected opposing contributions from a small number of foods. Reducing UPF consumption can generate substantial health and economic benefits, but environmental co-benefits are not automatic and depend on the dietary patterns that replace UPFs.

## 1. Introduction

Unhealthy diets and the environmental pressures generated by food production are increasingly interconnected global challenges. Food systems are a major source of environmental degradation, accounting for approximately 26% of anthropogenic greenhouse gas emissions, 32% of global terrestrial acidification, 66% of freshwater withdrawals, and 78% of marine and freshwater eutrophication [1, 2]. At the same time, the availability of ultra-processed foods (UPFs) has expanded rapidly worldwide, particularly in middle-income countries undergoing dietary transitions [3]. This combination raises an important policy question: whether reducing UPF consumption can improve population health while also contributing to more environmentally sustainable diets.

UPFs, as defined by the NOVA classification, are industrial formulations made largely from food-derived substances and cosmetic additives, with little or no intact whole foods [4, 5]. Evidence from observational and experimental studies indicates that their increasing contribution to the diet has important health consequences. Umbrella and dose–response meta-analyses consistently associate higher UPF consumption with cardiometabolic outcomes and all-cause mortality [6, 7]. In a controlled inpatient crossover trial, an ultra-processed diet increased ad libitum energy intake and body weight compared with an unprocessed diet matched for presented energy, macronutrients, fibre, sodium and sugar [8]. These findings are consistent with proposed mechanisms involving altered eating rate and satiety signalling, high glycaemic loads, changes in the food matrix, and exposure to additives and packaging-related chemicals [5, 6, 9].

The environmental implications of UPF consumption have received less attention and the available evidence focuses mainly on the footprint of observed diets. In France, for example, UPFs represented 19% of the diet but contributed 24% of diet-related greenhouse gas emissions, 23% of water and land use, and 26% of energy demand [10]. A systematic review of 52 studies similarly found that UPFs accounted for up to one-third of diet-related greenhouse gas emissions, land use and food waste, and up to one-quarter of diet-related water use among adults in several high-income countries [11]. These studies show that UPFs constitute a relevant component of the environmental footprint of contemporary diets, in addition to their contribution to adverse health outcomes.

However, evidence on the footprint of observed UPF-rich diets does not establish what happens when UPF consumption is reduced and the displaced foods or energy are replaced. Although UPFs can generate additional impacts through processing, packaging and long supply chains [12], replacing them with less processed foods does not necessarily improve all environmental indicators simultaneously [10, 11, 13]. The environmental outcome will also depend on the food-group composition and environmental intensity of the replacement basket. Similarly, comparative risk assessment (CRA) studies have estimated mortality attributable to UPF consumption and avoidable deaths in Brazil and other countries [14, 15], but have not examined whether these health gains are accompanied by environmental co-benefits under alternative substitution patterns. Argentina provides a relevant setting in which to examine this question because UPFs contribute substantially to national dietary energy intake [16].

To address this gap, we propose an evaluation framework to assess health-environment trade-offs under alternative UPF-reduction policy scenarios. Using Argentina as a policy-relevant case study, we estimate the mortality burden and mortality-related productivity losses attributable to UPF consumption and evaluate how greenhouse gas emissions, land use, water use and freshwater eutrophication change under alternative replacement scenarios. By linking CRA with scenario-based environmental modelling, our approach extends beyond burden estimation and whole-basket footprint comparisons. It separates the consequences of reducing UPF exposure from those of the replacement pattern and identifies the food groups responsible for environmental trade-offs within a guideline-concordant basket. We ask whether environmental co-benefits accompany the health gains of UPF reduction, how they depend on substitution design, and which food groups account for the resulting trade-offs.

## 2. Methods

### 2.1. Overview and study population

**Figure 1.**
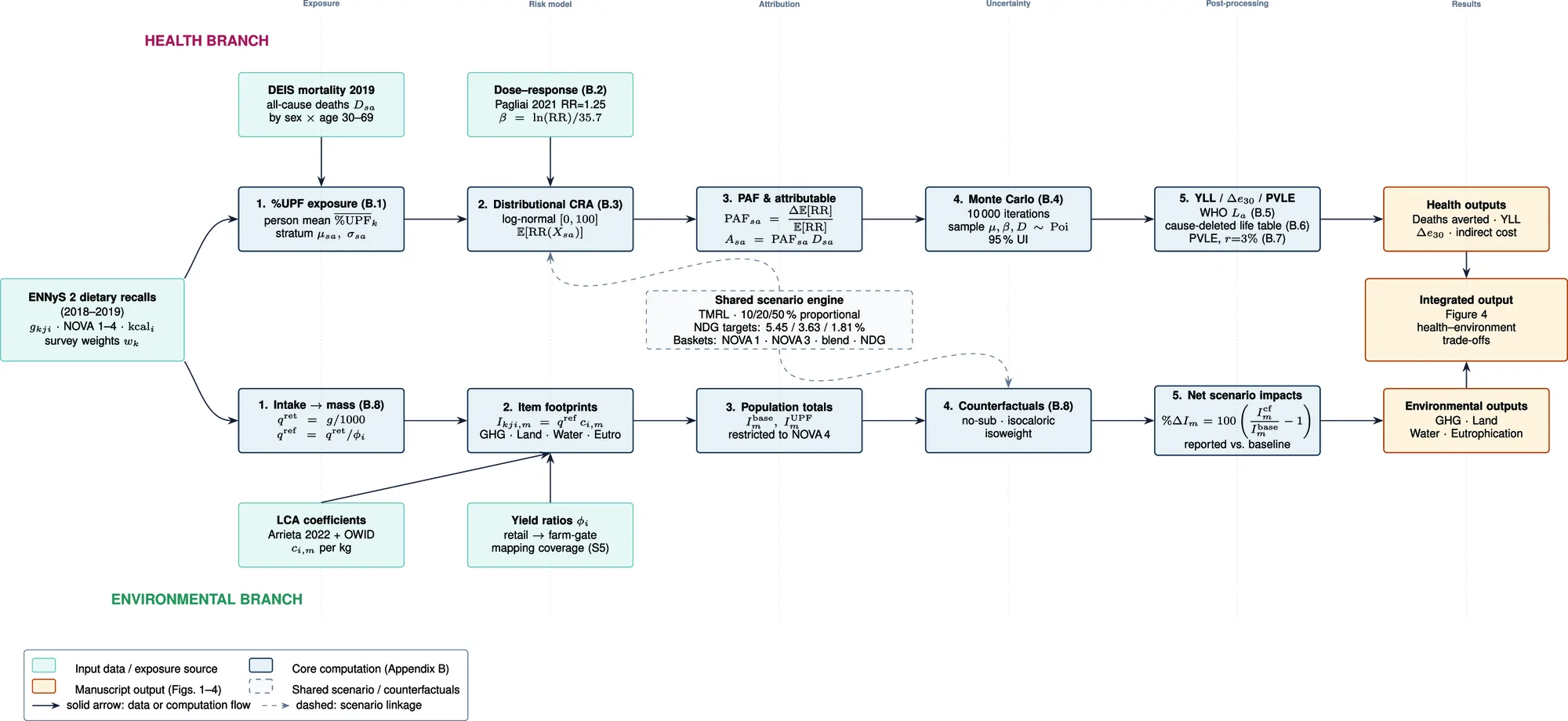
Overview of the health and environmental analytical workflows. *Note.* The left branch summarises the health module, linking dietary exposure from Argentina’s Second National Survey on Nutrition and Health (ENNyS 2), mortality counts, and the comparative risk assessment framework to deaths attributable to ultra-processed foods, years of life lost, life expectancy, and indirect economic costs. The right branch summarises the environmental module, linking ENNyS 2 food intakes to mapped environmental coefficients and counterfactual replacement structures to estimate baseline UPF shares and net changes in greenhouse gas emissions, land use, water use, and freshwater eutrophication. The central scenario engine applies the same TMRL, proportional-reduction, NDGs-target, and replacement-basket definitions across both branches, and labels B.1–B.8 refer to the corresponding sections of Online appendix B.

We developed a CRA macrosimulation [17–19] to quantify the mortality burden attributable to UPF consumption among Argentine adults and to estimate years of life lost (YLL), gains in life expectancy, indirect economic costs, and environmental impacts under policy-relevant UPF-reduction scenarios. The model linked nationally representative dietary exposure distributions to mortality counts by sex and five-year age group, and propagated uncertainty through Monte Carlo simulation. An overview of the health and environmental analytical workflows is shown in figure 1.

Dietary exposure was obtained from Argentina’s Second National Survey on Nutrition and Health (ENNyS 2), a cross-sectional, multistage, region-stratified probabilistic survey conducted in Argentine urban localities in 2018–2019. References [20, 21] Dietary intake was collected using 24 h recalls with a multiple-pass approach [20]. We restricted the analysis to adults aged 30–69 years to focus on premature mortality from diet-related chronic diseases. In the analytic age range, 4496 adults had at least one linked dietary recall with food-intake information, and repeated recalls were available for 1011 participants. Full methodological details, including life table construction, uncertainty propagation, environmental accounting and replacement-basket construction, are provided in the Online appendix.

### 2.2. Dietary exposure and UPF contribution to total energy

All reported foods and beverages were classified according to the NOVA framework [4]. We operationalised UPF exposure as the percentage of total dietary energy derived from NOVA group 4 items (%UPF). For each 24 h recall, UPF energy was computed from item-level grams and official kcal/100 g values from the ENNyS 2/SARA 2 composition resources [22], and divided by the official total recall energy:

\begin{align*} \%\mathrm{UPF}_{kj} \; = \; 100 \times \frac{E^{\mathrm{UPF}}_{kj}}{E^{\mathrm{tot}}_{kj}},\end{align*}

where $E^{\mathrm{UPF}}_{kj}$ includes only UPF items with available energy density and $E^{\mathrm{tot}}_{kj}$ is the official total energy for recall j of participant k. Recalls with non-positive total energy or values outside the feasible range were excluded. For participants with repeated recalls, recall-level %UPF was averaged within person before estimation of stratum-specific exposure distributions. The item-level classification is reported in Online appendix table S2 and the overall NOVA-group distribution in Online appendix table S3.

We estimated sex- and age-specific exposure distributions for eight five-year age groups (30–34 to 65–69 years) using calibrated ENNyS 2 survey weights. For CRA calculations, exposure in each stratum was represented by a log-normal distribution parameterised from the weighted mean and standard deviation of %UPF and evaluated on the bounded support [0,100] (Online appendix, section B.1).

### 2.3. Mortality outcomes

Premature deaths were defined using all-cause mortality and classified by underlying cause according to the 10th revision of the International Statistical Classification of Diseases (ICD-10). We considered all deaths coded A00–Y89. Data on deaths in 2019 were obtained from Argentina’s Directorate of Health Statistics and Information (DEIS) [23] and aggregated by sex and the same eight five-year age groups used in the dietary analysis, yielding 16 analytic strata. The all-cause mortality input for the CRA model comprised 103 448 deaths among adults aged 30–69 years.

### 2.4. Counterfactual UPF-reduction scenarios

Counterfactual scenarios were defined as shifts in the %UPF distribution within each sex–age stratum. We pre-specified two families of scenarios to span theoretical, proportional and policy-anchored contrasts.

**Theoretical and proportional scenarios.** The theoretical minimum risk level (TMRL) set %UPF to approximately zero for all individuals and served as the attributable-burden reference under CRA convention. Proportional reduction scenarios applied uniform relative decreases of 10%, 20% and 50% to baseline %UPF within each stratum.

**Guideline-aligned scenarios.** To reflect plausible policy targets grounded in Argentine dietary guidance, we implemented scenarios based on the food-group structure of Argentina’s National Dietary Guidelines (NDGs). Ministerio de Salud de la Nacion Argentina [24] The basic idea was to define a NDGs-concordant diet in which each food group was allowed to contain only a small residual share of UPFs. For example, groups that are normatively expected to be consumed in minimally processed form were assigned very low or zero residual UPF content, whereas groups in which some processed products are more plausibly consistent with the guideline were allowed a modest residual share. These group-specific assumptions were then aggregated using the normative NDGs basket composition to obtain an overall target for the percentage of total dietary energy derived from UPFs.

For each NDGs scenario profile ($q \in \{\mathrm{conservative}, \mathrm{central}, \mathrm{strict}\}$), the target was computed as

\begin{align*} \%\mathrm{UPF}^{\mathrm{target}}_q = 100\sum_g w_g\,c_{gq},\end{align*}

where $w_g$ is the normalised NDGs basket composition weight assigned to food group g (used as an energy-proxy weight, with $\sum_g w_g = 1$), and $c_{gq}$ is the residual UPF cap assigned to that food group under profile q. Thus, the overall target is a weighted average of the maximum UPF shares allowed across food groups, with weights given by the guideline-recommended composition of the diet.

The central profile represents our main set of food-group-specific residual UPF caps used to operationalise a guideline-concordant diet. Conservative and strict profiles were defined by applying an exploratory $\pm50\%$ range around those central caps, thereby allowing a somewhat higher or lower residual UPF content while preserving the same underlying NDGs food-group structure. This yielded target %UPF values of 5.45 (conservative), 3.63 (central), and 1.81 (strict). Full construction details, including the group-specific caps and the derivation of the central target, are provided in the Online appendix (section B.3, table B1).

### 2.5. Risk function and CRA

We treated hazard ratios from the source meta-analysis as approximations to relative risks (RR), consistent with standard CRA practice when outcome incidence is low relative to the study population [17]. UPF exposure was associated with all-cause mortality using a log-linear dose–response function:

\begin{align*} \mathrm{RR}\left(p\right) \; = \; \exp\left(\beta \cdot p\right), \qquad \beta \; = \; \frac{\ln\left(\mathrm{RR}_{\mathrm{ref}}\right)}{x_{\mathrm{ref}}},\end{align*}

where p is the share of dietary energy from UPFs (percentage points, 0–100), $\mathrm{RR}_{\mathrm{ref}}$ is the reference RR from meta-analysis, and $x_{\mathrm{ref}}$ is the exposure contrast used to calibrate the slope in this CRA implementation. We used $\mathrm{RR}_{\mathrm{ref}} = 1.25$ (95% CI 1.14–1.37) from Pagliai *et al* [25]. and, following prior UPF CRA applications [14], calibrated β by mapping this RR to $x_{\mathrm{ref}} = 35.7$ percentage points (an approximately 0%–35.7% contrast). Thus, $x_{\mathrm{ref}}$ should be interpreted as a calibration parameter rather than a universal threshold.

For each sex–age stratum, we computed the population attributable fraction (PAF) using a distributional CRA approach that integrates the RR over the full exposure distribution rather than evaluating it only at the mean:

\begin{align*} \mathrm{PAF} \; = \; \frac{\mathrm{E}\left[\mathrm{RR}\left(P\right)\right] \;-\; \mathrm{E}\left[\mathrm{RR}\left(P^{\mathrm{cf}}\right)\right]}{\mathrm{E}\left[\mathrm{RR}\left(P\right)\right]},\end{align*}

where P and $P^{\mathrm{cf}}$ are the baseline and counterfactual exposure distributions, respectively. Because %UPF is bounded, expectations were evaluated over [0,100] using a log-normal density and a fine discretisation grid. Attributable deaths were computed as $D_{\mathrm{att}} = \mathrm{PAF} \times D$, where D is the observed number of deaths in the stratum. For proportional scenarios, the counterfactual exposure distribution was scaled directly; for NDGs-aligned scenarios, the counterfactual mean was capped at the scenario target and the standard deviation was scaled proportionally before recomputing expected risk.

Uncertainty was quantified by Monte Carlo simulation (10 000 iterations). In each iteration we jointly sampled (i) stratum-specific mean %UPF using a normal approximation based on survey-derived standard errors, (ii) the dose–response slope β using a normal approximation on the log-risk scale, and (iii) death counts using a Poisson distribution. All results are reported as medians with 95% uncertainty intervals (2.5th and 97.5th percentiles).

### 2.6. YLL, life expectancy, and indirect economic costs

YLL were calculated by multiplying stratum-specific attributable deaths by standard expected remaining years of life at the age-group midpoint, taken from the WHO reference life table [26]. Life expectancy gains were estimated using abridged cause-deleted life tables in which UPF-attributable deaths were removed from each sex–age stratum and age-specific mortality rates were recomputed under the same life-table assumptions, including a terminal-life assumption for the final age interval (Online appendix, section B.6).

Indirect economic costs were estimated with a human capital approach. We valued each premature death as the present value of lifetime earnings lost (PVLE), i.e. forgone market earnings from the age-group midpoint to an assumed retirement age of 65 years for both sexes, using sex- and age-specific employment rates and labour income from Argentina’s Permanent Household Survey (EPH, 2019, fourth quarter). References [27, 28] In Argentina, statutory retirement ages are generally 60 years for women and 65 years for men; we used a uniform ag$10^{-65}$ horizon as a simplifying modelling assumption to maintain a common valuation horizon across strata [29]. Future earnings were discounted at 3% annually, consistent with standard economic-evaluation reference-case practice [30, 31]. All costs were converted to international dollars using the World Bank purchasing power parity (PPP) conversion factor for Argentina in 2019. World Bank [32] Under this specification, deaths in the 65–69 years group do not generate additional labour-market losses. Stratum-specific PVLE estimates are reported in Online appendix table S4.

The economic estimates were restricted to productivity losses resulting from premature mortality before the assumed retirement age. They did not include direct health-care expenditure or productivity losses associated with non-fatal illness, including sickness absence, reduced productivity while working, disability or early labour-force exit.

### 2.7. Environmental impacts and scenarios

Environmental impacts were estimated by linking ENNyS 2 food intake records to food-group-specific life-cycle assessment coefficients for greenhouse gas emissions, land use, water use, and freshwater eutrophication, expressed per kilogram of product at a harmonised reference system. Coefficients prioritised Argentina-focused evidence [13, 33, 34] and were complemented with global datasets when direct matches were unavailable [1, 35, 36]. Because the environmental coefficients were defined at the level of food groups rather than individual ENNyS food codes, reported intake records were first mapped to an environmental grouping system. Multiple ENNyS food codes could therefore share the same environmental coefficient set. Where the physical reference underlying a coefficient differed from the observed intake mass, consumed grams were converted to the appropriate reference mass before applying the impact factor. Mapping coverage is reported in Online appendix table S5 and the coefficient set used in the manuscript specification in Online appendix table S8. Plain drinking water and ice were excluded from the environmental scope because they contribute negligible dietary energy and would otherwise dominate mass-based coverage summaries.

For each individual k, recall day j, food code i, and environmental indicator m, item-level impact was calculated as

\begin{align*} I_{kji,m} = q_{kji}^{\mathrm{ref}}\,c_{i,m},\end{align*}

where $q_{kji}^{\mathrm{ref}}$ is the food quantity expressed in the reference mass required by the coefficient and $c_{i,m}$ is the corresponding impact intensity. Baseline totals were then obtained by summing item-level impacts over all in-scope foods and weighted records:

\begin{align*} I_m^{\mathrm{base}} = \sum_{k,j}\sum_i I_{kji,m}.\end{align*}

The UPF-attributable component was calculated by restricting the same accounting framework to foods classified as NOVA 4. This allowed us to quantify both the contribution of UPFs to observed dietary impacts and the net change in impacts under counterfactual reductions in UPF consumption.

Environmental counterfactuals mirrored the same six health scenarios but differed in what happened after the UPF-attributable component was removed. We evaluated three substitution designs: no substitution, isocaloric substitution, and isoweight substitution. In all cases, the first step was to remove a specified fraction of the environmental burden attributable to UPFs. The gross removed impact for indicator m was therefore

\begin{align*} I_m^{\mathrm{rem}} = r\,I_m^{\mathrm{UPF}},\end{align*}

where r is the proportional reduction implied by the scenario. The key difference across these three substitution designs lies in whether the removed UPF burden is not replaced at all, replaced on an energy-equivalent basis, or replaced on a mass-equivalent basis.

**No substitution.** A fraction of the UPF component was removed and not replaced, reducing both total dietary energy and total dietary mass. The counterfactual therefore becomes

\begin{align*} I_m^{\mathrm{cf}} = I_m^{\mathrm{base}} - r\,I_m^{\mathrm{UPF}}.\end{align*}

This scenario can be interpreted as a mechanical upper bound for environmental savings, because the removed UPF burden is not offset by any replacement foods.

**Isocaloric substitution.** Energy removed by reducing UPFs was replaced with non-UPF foods so that total dietary energy was preserved. Replacement was based on basket-specific environmental intensities expressed per kilocalorie. We considered four substitution baskets: NOVA 1 foods, NOVA 3 foods, a weighted NOVA 1/NOVA 3 mix, and an NDGs-concordant basket aligned with the Argentine NDGs [24]. The NOVA 1 and NOVA 3 baskets were derived from the observed composition of mapped non-UPF foods in those respective NOVA classes, the weighted mix interpolated between those two structures using their observed mapped energy shares, and the NDGs basket used food-group weights implied by the NDGs. This design allowed the environmental consequences of UPF reduction to depend explicitly on the composition of the substitution basket.

The NDGs-concordant basket was constructed as a model-based operationalisation of the Argentine NDGs rather than as a set of percentages stated verbatim in the guideline. Briefly, the broad NDGs food-guide structure was translated into the environmental food-group classification used in our model. Aggregate guideline categories for fruits and vegetables, legumes/cereals/starchy foods, dairy, animal-source foods, and oils/nuts/seeds were mapped onto the 18 environmental groups using the guideline food plan, quantitative recommendations, and qualitative guidance on food-group composition. Within the fruit-and-vegetable block, weights were partitioned according to the recommended daily quantities of fruit and vegetables, while the remaining groups were apportioned using recommended daily quantities and qualitative guidance on dairy servings, fish frequency, moderate red-meat intake, and oils/nuts/seeds. The resulting NDGs-concordant basket should therefore be interpreted as a guideline-based modelling assumption used to define replacement-food intensities rather than as a direct reproduction of fixed guideline percentages. Full details of the replacement-basket construction are provided in the Online appendix, section B.8, and the resulting basket composition is reported in Online appendix table S7.

**Isoweight substitution.** Removed UPF mass (kg) was replaced one-for-one with non-UPF foods using the same set of replacement baskets, with impacts calculated from basket-specific environmental intensities per kilogram rather than per kilocalorie. This specification serves as a sensitivity analysis, because it assumes replacement on a mass basis rather than on an energy-equivalent basis. Comparing isocaloric and isoweight scenarios therefore helps assess the extent to which environmental results depend on the accounting unit used for substitution.

The environmental module was designed to isolate substitution effects rather than broader food-system adjustment. It therefore assumes that dietary-demand shifts are met within the same production system represented by the mapped coefficients and does not explicitly model imports, technological adaptation, price responses, or market feedbacks. Basket composition, scenario-specific environmental results and food-group contributions are summarised in Online appendix tables S6–S9 and figure S1.

**Food-group decomposition.** The scenarios described above summarise the net environmental change associated with each replacement basket, but they do not identify the foods producing that change. We therefore decomposed the strict NDGs-concordant isocaloric scenario into food-group-specific contributions. For food group g and environmental indicator m, the contribution was calculated as

\begin{align*} \Delta I_{g,m} = r\,E^{\mathrm{UPF}}s_g\theta_{g,m}-r\,I^{\mathrm{UPF}}_{g,m},\end{align*}

where $E^{\mathrm{UPF}}$ is the aggregate baseline energy from mapped UPFs, $s_g$ is the energy share of food group g in the replacement basket, $\theta_{g,m}$ is the environmental impact intensity per kilocalorie of group g for indicator m, $I^{\mathrm{UPF}}_{g,m}$ is the baseline impact for indicator m of the UPFs mapped to group g, and r is the scenario-specific reduction. The first term represents the impact added through substitution and the second the impact removed with the displaced UPFs. Thus, each contribution is a net quantity and the contributions add up to the aggregate scenario result by construction, $\sum_g\Delta I_{g,m} = \Delta I_m$. Contributions were expressed as a percentage of the corresponding baseline dietary impact. Food-group results are reported in Online appendix section B.8, table S9 and figure S1.

## 3. Results

**Figure 2.**
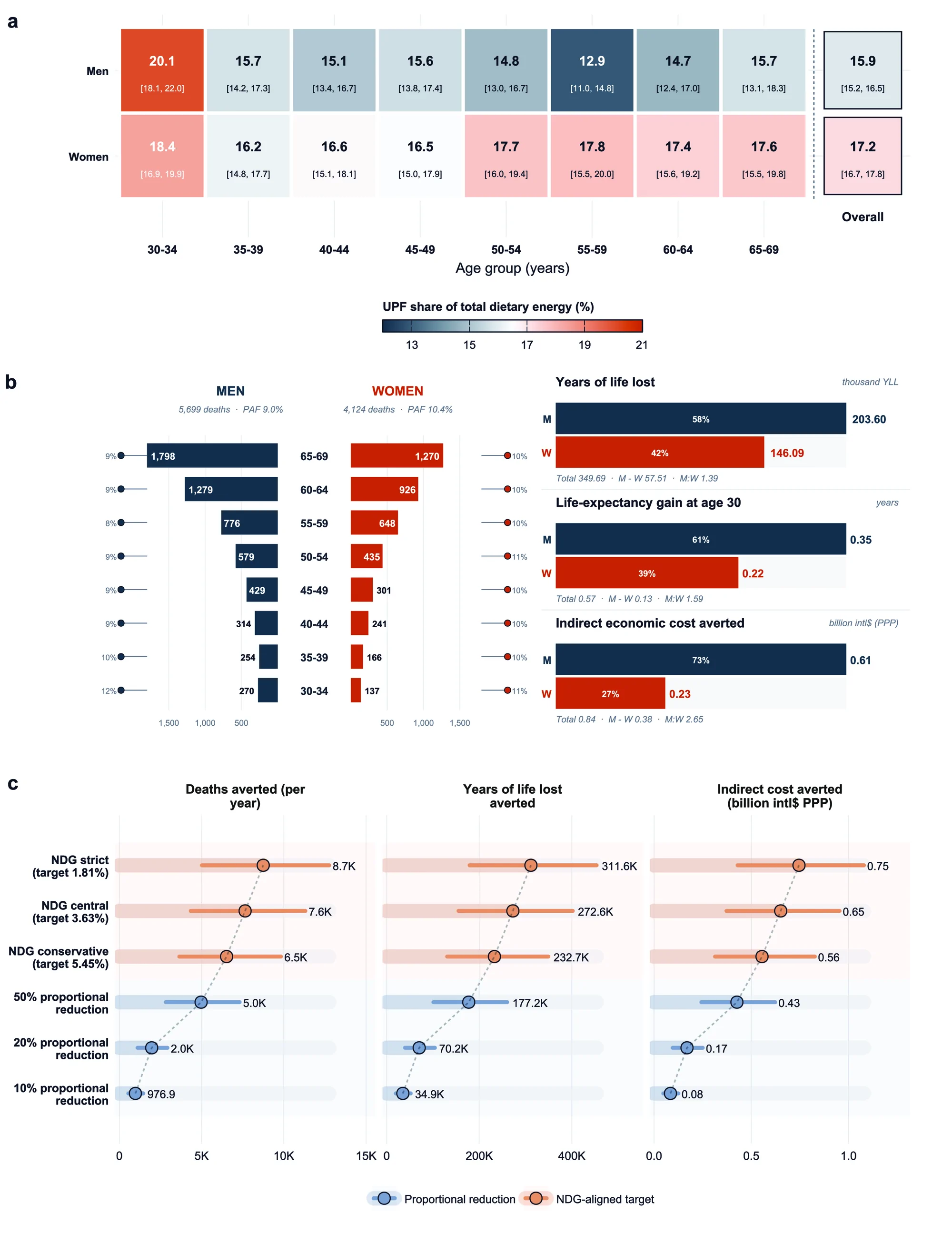
Ultra-processed food exposure, attributable burden, and gains under reduction scenarios in Argentine adults aged 30–69 years. *Note.* Panel a shows the mean share of total dietary energy derived from ultra-processed foods (UPFs) by sex and 5 year age group, with an additional overall column reporting the age-standardised mean for each sex. Values inside cells are percentages and brackets indicate 95% confidence intervals. Panel b shows UPF-attributable deaths in 2019 by sex and age group, together with stratum-specific population attributable fractions; the right-hand side of the same panel summarises years of life lost, gains in life expectancy at age 30, and indirect economic costs by sex. Panel c shows deaths averted, years of life lost averted, and indirect costs averted under proportional and NDG-aligned reduction scenarios; points denote central estimates and horizontal lines indicate 95% uncertainty intervals. NDG denotes National Dietary Guidelines, PAF population attributable fraction, YLL years of life lost, PPP purchasing power parity, and UPF ultra-processed food.

UPF consumption accounted for a substantial share of total dietary energy across all sex–age strata among Argentine adults aged 30–69 years (figure 2(a)). Mean values ranged from 12.9% to 20.1% in men and from 16.2% to 18.4% in women, with women generally showing higher exposure across age groups. The youngest group included in the analysis (30–34 years) had the highest mean UPF share in both sexes, at 20.1% in men and 18.4% in women. In the oldest group (65–69 years), the corresponding values were 15.7% and 17.6%, representing cross-sectional differences of 4.4% and 0.8%, respectively. However, the intermediate age groups did not show a monotonic age gradient. Overall age-standardised means were 15.9% in men and 17.2% in women.

In 2019, this exposure was associated with 9823 attributable deaths, including 5699 in men and 4124 in women (figure 2(b)). Attributable deaths increased with age in both sexes, peaking at 1798 in men and 1270 in women in the 65–69 years age group. Mean population attributable fractions were 9.0% in men and 10.4% in women, indicating a higher relative burden in women but a larger absolute burden in men. Overall, UPF consumption was associated with 349.69 thousand YLL, gains in life expectancy at age 30 of 0.35 years in men and 0.22 years in women, and indirect economic costs of 0.84 billion international dollars (2019 PPP).

Counterfactual reductions in UPF consumption generated substantial and graded health and economic gains (figure 2(c)). A 10% proportional reduction was associated with 976.9 deaths averted per year, 34.9 thousand YLL averted, and 0.08 billion international dollars in indirect costs averted. These gains increased progressively under more ambitious scenarios, reaching 5.0 thousand deaths averted, 177.2 thousand YLL averted, and 0.43 billion international dollars under a 50% reduction. NDGs-aligned targets produced the largest gains, from 6.5 thousand deaths averted and 0.56 billion international dollars under the conservative target to 8.7 thousand deaths averted and 0.75 billion under the strict target, approaching nine-tenths of the total modelled attributable burden.

**Figure 3.**
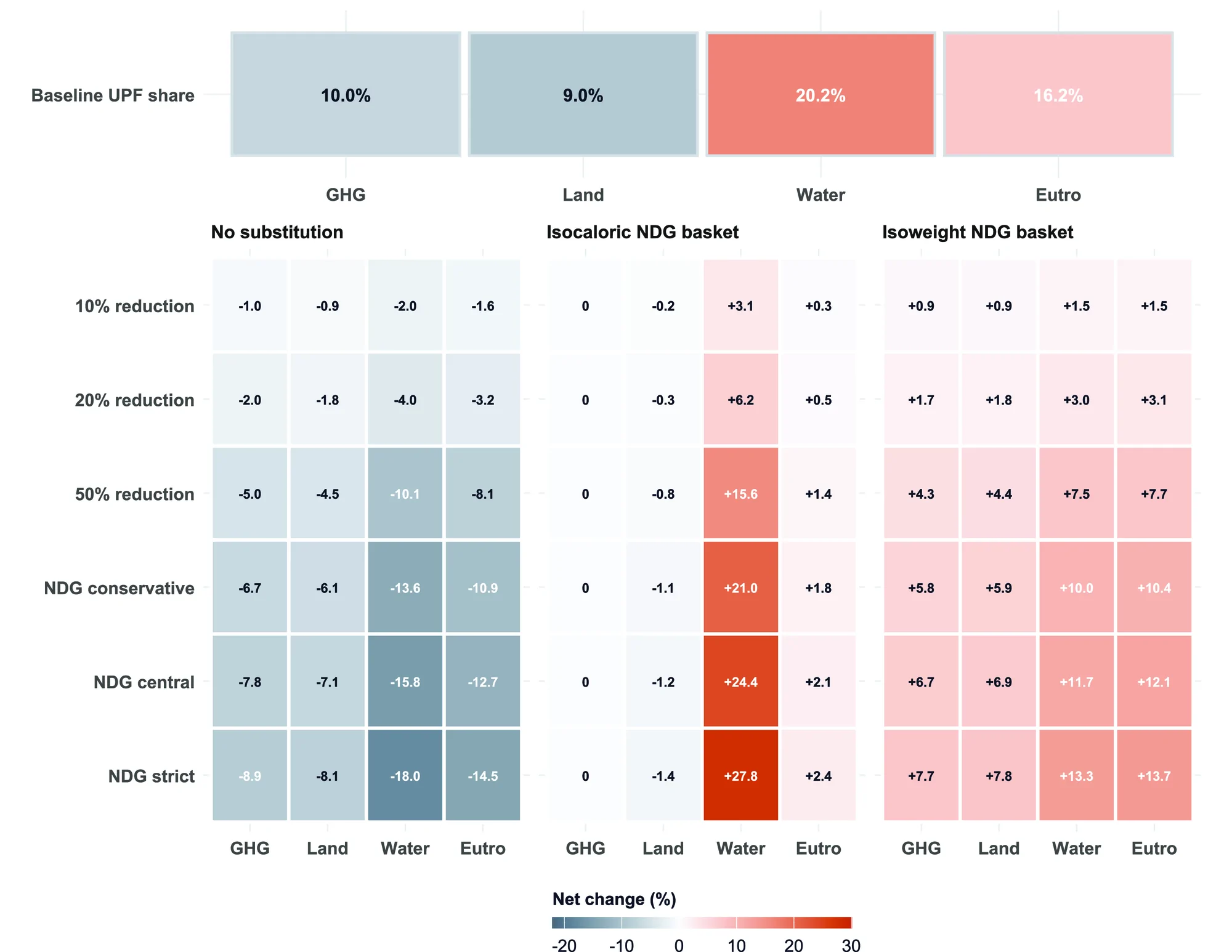
Environmental implications of reducing ultra-processed food consumption under alternative substitution designs. *Note.* The top strip shows the baseline share of each mapped environmental indicator attributable to UPFs. The three lower heatmaps show the net percentage change versus baseline across the six UPF-reduction scenarios under no substitution, isocaloric substitution with the NDG basket, and isoweight substitution with the same basket. Rows correspond to scenario intensity and columns to greenhouse gas emissions, land use, water use, and freshwater eutrophication. Negative values indicate reductions and positive values indicate increases relative to the baseline diet. GHG denotes greenhouse gas emissions, Eutro freshwater eutrophication, NDG National Dietary Guidelines, and UPF ultra-processed food.

Environmental outcomes diverged sharply across substitution scenarios (figure 3). At baseline, UPFs accounted for 10.0% of mapped greenhouse gas emissions, 9.0% of land use, 20.2% of water use and 16.2% of freshwater eutrophication. Under no substitution, all four indicators declined monotonically as reduction intensified, reaching −8.9% for greenhouse gas emissions, −8.1% for land use, −18.0% for water use and −14.5% for eutrophication in the NDG strict scenario. By contrast, under isocaloric substitution with the NDG-concordant basket, greenhouse gas emissions remained close to zero and land use declined only slightly, whereas water use increased sharply, up to +27.8%, and eutrophication rose modestly, up to +2.4%. Under isoweight substitution with the same basket, all four indicators increased, reaching +7.7%, +7.8%, +13.3% and +13.7%, respectively. These results indicate that the environmental consequences of UPF reduction depended critically on the substitution scenario rather than on the scale of reduction alone.

**Figure 4.**
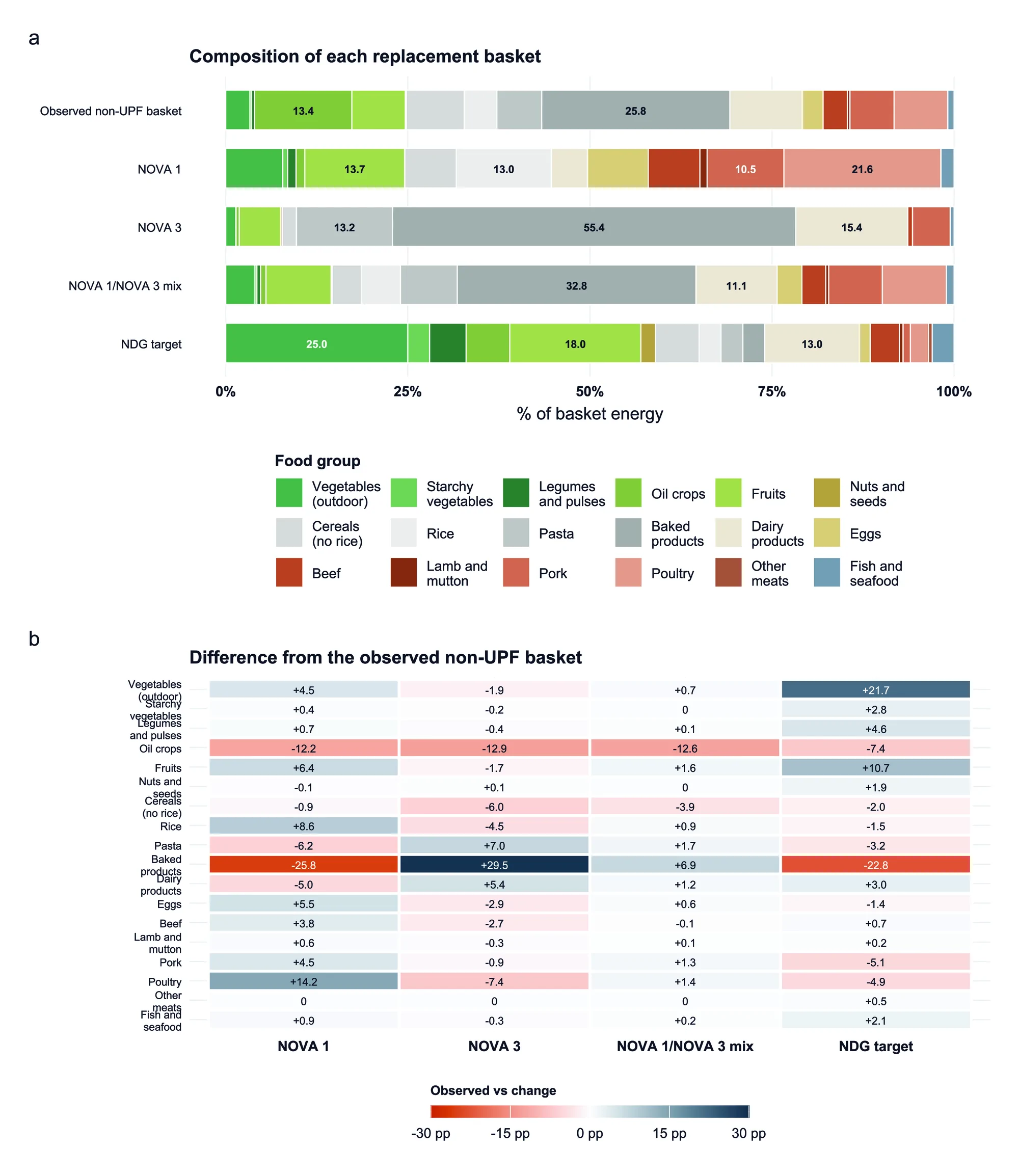
Composition of the replacement baskets used in the environmental scenarios. *Note.* Panel a shows the percentage contribution of each food group to total basket energy in the observed non-UPF basket, defined as the empirical basket of mapped non-UPF foods consumed in ENNyS 2, together with the NOVA 1 basket, the NOVA 3 basket, the NOVA 1/NOVA 3 mix, and the NDG-concordant basket. Labels are shown for selected food-group shares to facilitate comparison across baskets. Panel b shows the percentage-point difference in each food-group share relative to the observed non-UPF basket; positive values indicate a larger contribution than in the observed non-UPF basket and negative values indicate a smaller one. Detailed basket composition is reported in Online appendix table S7. NDG denotes National Dietary Guidelines, and UPF denotes ultra-processed food.

Differences between scenarios were closely linked to the composition of the substitution baskets. Figure 3 defines the counterfactual reduction and substitution scenarios, whereas figure 4 shows the resulting dietary compositions implied by those scenarios. This step is essential because the health and environmental consequences of lowering UPF consumption depend not only on the magnitude of the reduction but also on the composition of the replacement diet.

Thus, the key contrast of the results in figure 3 was not simply between more and less processed substitutes, but between distinct food-group mixes with different environmental intensities per kilocalorie. Compared with the foods that currently make up the non-UPF portion of the diet, the NDG-concordant basket allocated larger shares to vegetables, fruits, legumes and dairy, while reducing baked products and oil crops. This shift helps explain why the NDG basket generated only small changes in greenhouse gas emissions and land use, yet substantial increases in water use and modest increases in eutrophication under isocaloric substitution. By contrast, the NOVA 3 basket remained much more concentrated in baked products and dairy, producing a different environmental profile and avoiding the large water-use penalty observed for the NDG basket. These results indicate that the environmental effects of UPF reduction were driven less by the act of removing UPFs itself than by the specific foods used to replace them.

Although figure 4 shows how the replacement baskets differ in their food-group composition, it does not indicate how much each group contributes to the resulting environmental changes. We therefore decomposed the strict NDG-concordant isocaloric scenario into net food-group contributions, defined as the environmental impact added through replacement minus the impact removed with the UPFs mapped to the same group (Online appendix table S9 and figure S1). Under this scenario, the increase in water use was driven primarily by outdoor vegetables, which contributed +26.1% of baseline water use, followed by cereals other than rice (+2.2%) and fruit (+0.9%). The near-zero aggregate change in greenhouse gas emissions concealed larger contributions of opposite sign; the increase associated with outdoor vegetables (+2.3%) was largely offset by reductions associated with beef (−1.8%), soft drinks (−0.6%) and coffee (−0.6%). The reduction in land use was driven mainly by beef (−2.1%), whereas the increase in eutrophication was associated primarily with other meats (+2.1%), outdoor vegetables (+1.5%) and lamb and mutton (+1.4%). Thus, relatively small aggregate changes can result from substantial offsetting contributions, and the food groups driving the results differ across environmental indicators.

**Figure 5.**
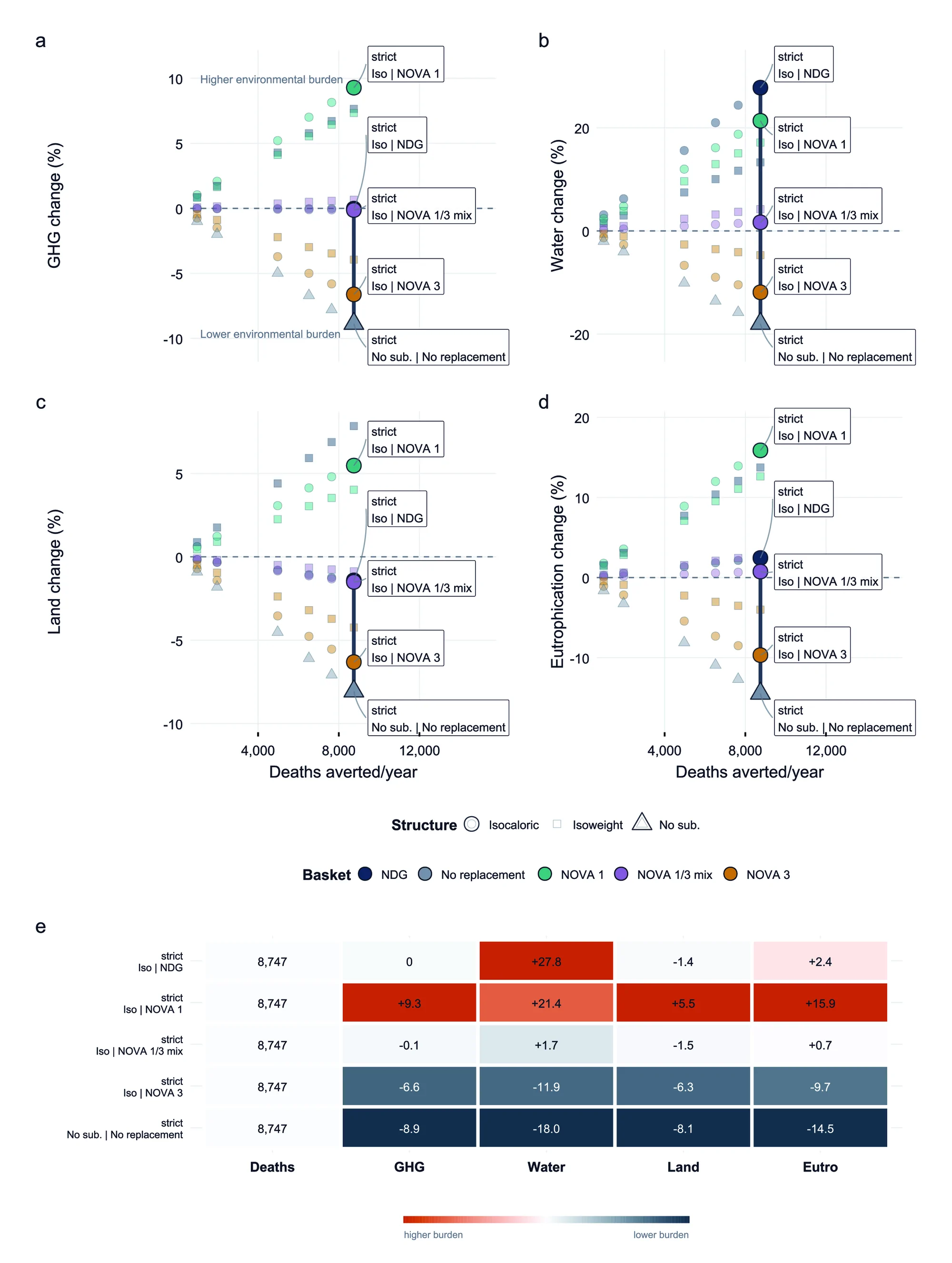
Health–environment trade-offs across UPF-reduction scenarios and replacement baskets. *Note.* Panels a–d plot annual deaths averted against the net percentage change versus baseline in greenhouse gas emissions, water use, land use, and freshwater eutrophication for each scenario–structure–basket combination. Point colour denotes replacement basket and point shape denotes substitution structure. The highlighted scenarios are those not jointly outperformed by any other combination across deaths averted and the four environmental indicators. Panel e summarises the outcome values for this highlighted set, showing deaths averted and the corresponding environmental changes side by side. NDGs denotes National Dietary Guidelines.

Figure 5 makes clear that similar health gains could be achieved through markedly different substitution scenarios. Under the strict target, both the no-substitution scenario and the isocaloric NOVA 3 substitution basket were associated with 8747 deaths averted per year while reducing greenhouse gas emissions, land use, water use and eutrophication. However, this alignment of health and environmental gains did not hold across substitution scenarios. The strict isocaloric NDG basket delivered the same reduction in deaths, but did so with a substantial increase in water use and a modest increase in eutrophication, whereas the strict isocaloric NOVA 1 basket increased all four environmental indicators despite achieving the same health gain. The mixed NOVA 1/NOVA 3 basket occupied an intermediate position, with near-zero greenhouse gas change, modest land savings, but increases in water use and eutrophication. These comparisons show that the health effects of reducing UPFs were determined primarily by the size of the reduction, whereas the environmental consequences depended on the composition of the substitution basket.

## 4. Discussion

Our findings show that reducing UPF consumption can generate substantial health gains, but environmental co-benefits are neither automatic nor uniform. Using nationally representative dietary data from Argentina, we found that UPF consumption was associated with a considerable burden of premature mortality, YLL and indirect economic costs, and that lowering UPF intake could avert an important share of this burden under policy-relevant scenarios. However, the environmental consequences depended strongly on the foods used to replace the displaced energy. Whereas the no-substitution scenarios reduced all four environmental indicators, the substitution scenarios produced more heterogeneous results, with some replacements improving certain dimensions while worsening others. Unlike assessments based only on the amount of UPFs removed or on the environmental footprint of the diet as a whole, our scenario design separates the effect of reducing UPF exposure from that of the replacement pattern. The results show that both dimensions need to be considered when evaluating the consequences of dietary change.

The estimated health burden is broadly consistent with previous evidence, although its magnitude reflects Argentina’s comparatively lower UPF consumption. Our estimated attributable fraction was slightly lower than recent CRA results for Brazil, where approximately 10.5% of premature deaths were attributed to UPF consumption [14]. This difference is consistent with the UPF energy share reported in ENNyS 2, which was 16.7% overall, 15.9% among men and 17.2% among women, and remains below that of the highest-consumption settings in the region. Our findings are also comparable to multi-country estimates combining national dietary surveys with meta-analytic risk functions, in which the attributable burden varies according to baseline UPF consumption and background mortality rates [15]. Differences across studies may also reflect modelling choices, including the exposure metric, age range, lag structure and availability of nationally representative dietary data.

Several mechanisms may explain why higher UPF consumption is associated with adverse health outcomes beyond differences in nutrient profiles. These include reduced satiety signalling, higher energy density and eating rate, alterations in the food matrix and glycaemic responses, and exposure to additives and packaging-related chemicals [6, 9]. Experimental evidence from a controlled feeding trial showed that, even when diets were matched on macronutrients, an ultra-processed diet increased ad libitum energy intake and short-term weight gain [8]. This evidence provides biological plausibility for at least part of the associations observed in epidemiological studies. Nevertheless, the RRs used in our analysis were pooled from observational cohort studies. In the source meta-analysis, four of the five contributing mortality studies reported multivariable adjustment, commonly including education or socioeconomic indicators, smoking, alcohol consumption, physical activity and BMI. However, covariate sets varied across studies; medication use was included in one contributing mortality cohort, whereas illicit drug use was not among the adjustment variables reported in the meta-analysis [25]. Residual confounding and measurement error may therefore remain, and the resulting burden estimates should be interpreted as modelled attributable burden under the stated CRA assumptions rather than as direct causal effects.

Beyond these health effects, the substitution scenarios show that reducing UPFs does not produce a single or uniformly favourable environmental outcome. Reductions in UPF intake generally lowered greenhouse gas emissions and land use in both the no-substitution and substitution scenarios, whereas the effects on water use and eutrophication were more sensitive to the replacement pattern. Under isocaloric substitution, some replacement baskets increased water use, showing that less processed or guideline-concordant foods are not necessarily lower-impact across all environmental dimensions. European cohort analyses have reported a related pattern, with higher UPF consumption associated with lower environmental impacts when measured per gram but higher impacts when measured per kilocalorie [37]. These differences reinforce the importance of considering more than one environmental indicator and of interpreting water-related results in the context of local production systems and water scarcity [11].

These aggregate results, however, do not show which food groups generated each environmental change or which groups offset it. To identify the contributions underlying these differences, we decomposed the strict NDGs-concordant isocaloric scenario into the environmental impact added by each replacement food group and the impact removed with the UPFs mapped to that same group. This analysis showed that outdoor vegetables were the main source of the increase in water use and also contributed to higher greenhouse gas emissions. For greenhouse gas emissions, however, this increase was largely offset by reductions associated with beef, soft drinks and coffee, resulting in a small aggregate change. The reduction in land use was driven mainly by beef, whereas the increase in eutrophication was associated mainly with other meats, outdoor vegetables and lamb and mutton. Thus, an apparently neutral aggregate effect may conceal substantial but opposing changes across food groups. Moreover, because the groups driving the results differed across indicators, the environmental implications of substitution cannot be inferred from a single food group or environmental dimension.

These indicator-specific trade-offs are particularly relevant when interpreting the guideline-concordant scenario. The basket derived from the NDGs delivered the full modelled health benefit, but its environmental profile was not uniformly favourable. Greenhouse gas emissions and land use changed little, while water use and eutrophication increased. Health-oriented dietary guidance cannot therefore be assumed to generate environmental co-benefits automatically. At the same time, these results should not be interpreted as an environmental assessment of the guidelines themselves. The decomposition shows that the outcome reflects the particular food-group composition used to operationalise the guidelines under our substitution scenario, rather than an inherent environmental property of the guidelines. Future revisions of dietary guidance, or complementary instruments implemented alongside it, could explicitly consider environmental criteria and assess health and environmental objectives jointly while preserving the nutritional goals for which the guidelines were developed.

From a policy perspective, three findings are particularly relevant. First, within our model, reducing UPF exposure generated the same health gains for a given reduction target irrespective of the composition of the replacement basket. Second, the environmental outcome depended on what replaced the displaced energy. Under the strict reduction target, replacement structures that averted the same 8747 deaths ranged from improving all four environmental indicators to worsening all four (figure 5). Third, the decomposition showed that these aggregate outcomes were often determined by a small number of food groups rather than by the replacement basket as a whole. Reducing UPF consumption alone is therefore not sufficient to improve the environmental profile of the diet. Policy evaluations should also examine which foods are consumed in place of UPFs.

Policy-induced substitutions are unlikely to be determined by consumers alone. Food manufacturers and retailers influence UPF exposure and the foods available as alternatives through product formulation, pricing, marketing, distribution and product portfolios [38, 39]. These margins can also change in response to regulation. Evidence indicates that firms respond to labelling, taxes and marketing restrictions through reformulation, strategic pricing and changes in marketing and product portfolios, although these responses can reinforce or weaken the intended policy effect [39, 40]. The role of government therefore extends beyond front-of-package labelling. In Chile, a package combining warning labels, marketing restrictions and school-food sales rules was followed by reductions in household purchases of calories and nutrients of concern, while evaluations also documented reformulation in several packaged-food categories [40, 41]. Fiscal measures can also affect demand, with implemented taxes associated with higher prices and lower sales of sugar-sweetened beverages [42]. Within the framework developed here, these interventions and the industry responses they trigger can affect both UPF exposure and the composition of the replacement diet.

These findings support a practical framework for evaluating candidate substitution strategies rather than recommending a particular regulatory instrument or replacement diet. Because our analysis does not estimate how individual interventions affect UPF consumption or the foods that replace it, candidate strategies could be assessed in three steps. First, the expected reduction in UPF exposure and the resulting replacement basket should be characterised separately. Second, the health gains associated with the reduction target should be estimated and the environmental consequences of the replacement basket compared across all four indicators. Third, the food-group decomposition could be used to identify which foods generate or offset the observed trade-offs, inform revisions of the basket and identify policy domains requiring further evaluation. In our results, the water-use contribution of outdoor vegetables points to production- and sourcing-side measures, including basin-specific water management, rather than to discouraging vegetable consumption [43]. The greenhouse gas and land-use savings associated with lower beef consumption highlight the relevance of replacement-basket and institutional menu composition [44], whereas the eutrophication contributions associated with lamb and other meats point to livestock nutrient and manure management [45]. These examples illustrate how the framework can narrow the range of interventions requiring subsequent evaluation, but their effects were not estimated in our model. Recommending specific instruments, replacement quantities or population-specific profiles would require additional information on production location and water scarcity, together with constraints on nutrient adequacy, affordability, cultural acceptability and realistic substitution behaviour, which were not incorporated into the present analysis.

This study has several strengths, but its findings should also be interpreted in light of important limitations. It used the most recent nationally representative dietary survey in Argentina, official mortality statistics, and a transparent CRA framework that was extended to YLL, life expectancy and indirect economic costs. We also modelled alternative UPF-reduction scenarios aligned with the NDGs and decomposed their environmental effects into food-group-specific contributions. However, the RRs were largely derived from cohort studies conducted in other settings and may not fully capture Argentina-specific confounding structures or effect modification. The CRA framework assumes immediate or short-term risk changes following dietary shifts and does not explicitly account for latency in chronic disease outcomes. The substitution scenarios necessarily simplify complex behavioural and market responses, and we did not jointly assess nutrient adequacy, affordability or acceptability. The environmental estimates are also subject to limitations. The food-group contributions inherit the resolution of the underlying environmental coefficients, which were obtained from international life-cycle assessment databases and, for some groups, relied on proxy values rather than Argentine production data. Individual contributions should therefore be interpreted as indicative of the relative importance of each food group rather than as precise magnitudes. In addition, the environmental module assumes that changes in dietary demand are met by domestic production and does not explicitly model imports. This assumption is unlikely to substantially affect the aggregate results because Food Balance Sheets indicate that imports account for a relatively small share of total food-energy availability in Argentina [46]. Nevertheless, import composition could still be relevant for particular food groups.

## 5. Conclusion

This study extends the assessment of UPFs beyond attributable mortality by showing that the consequences of reducing their consumption depend not only on the magnitude of the reduction, but also on the foods that replace them. In Argentina, UPF consumption was associated with a substantial modelled burden of premature mortality among adults aged 30–69 years, corresponding to 9823 deaths and 349 686 YLL in 2019 under CRA assumptions, with a larger burden among men than women. Achieving UPF-reduction targets aligned with the NDGs could generate important health and economic benefits. However, environmental outcomes varied markedly across replacement scenarios, and the food-group decomposition showed that aggregate changes could be driven by a small number of foods. Policies aimed at reducing UPF consumption should therefore be evaluated both by their capacity to reduce exposure and by the dietary patterns they are likely to induce. Future research should incorporate stronger evidence on substitution behaviours, locally calibrated environmental coefficients, nutrient adequacy, affordability and distributional effects across population groups, so that food policies can be assessed in terms of their broader health, economic and environmental consequences.

## Data Availability

The code is publicly available at: https://github.com/garciawitulski/UPFs_Health-Environment [47]. ENNyS 2 microdata are available through the Ministry of Health of Argentina. Mortality data are publicly available through the DEIS (https://datos.gob.ar). EPH microdata are available through INDEC (https://www.indec.gob.ar). Supplementary appendix available at https://doi.org/10.1088/1748-9326/aea587/data1.
